# The Kaiser Permanente Northern California Adult Alcohol Registry, an Electronic Health Records-Based Registry of Patients With Alcohol Problems: Development and Implementation

**DOI:** 10.2196/19081

**Published:** 2020-07-22

**Authors:** Vanessa A Palzes, Constance Weisner, Felicia W Chi, Andrea H Kline-Simon, Derek D Satre, Matthew E Hirschtritt, Murtuza Ghadiali, Stacy Sterling

**Affiliations:** 1 Division of Research Kaiser Permanente Northern California Oakland, CA United States; 2 Department of Psychiatry Weill Institute of Neurosciences University of California San Francisco, CA United States; 3 Department of Psychiatry Kaiser Permanente East Bay Oakland, CA United States; 4 Department of Addiction Medicine Kaiser Permanente San Francisco Medical Center San Francisco, CA United States; 5 Department of Addiction Psychiatry University of California San Francisco, CA United States

**Keywords:** electronic health records, alcohol, registry, unhealthy alcohol use, alcohol use disorder, recovery, secondary data

## Abstract

**Background:**

Electronic health record (EHR)–based disease registries have aided health care professionals and researchers in increasing their understanding of chronic illnesses, including identifying patients with (or at risk of developing) conditions and tracking treatment progress and recovery. Despite excessive alcohol use being a major contributor to the global burden of disease and disability, no registries of alcohol problems exist. EHR-based data in Kaiser Permanente Northern California (KPNC), an integrated health system that conducts systematic alcohol screening, which provides specialty addiction medicine treatment internally and has a membership of over 4 million members that are highly representative of the US population with access to care, provide a unique opportunity to develop such a registry.

**Objective:**

Our objectives were to describe the development and implementation of a protocol for assembling the KPNC Adult Alcohol Registry, which may be useful to other researchers and health systems, and to characterize the registry cohort descriptively, including underlying health conditions.

**Methods:**

Inclusion criteria were adult members with unhealthy alcohol use (using National Institute on Alcohol Abuse and Alcoholism guidelines), an alcohol use disorder (AUD) diagnosis, or an alcohol-related health problem between June 1, 2013, and May 31, 2019. We extracted patients’ longitudinal, multidimensional EHR data from 1 year before their date of eligibility through May 31, 2019, and conducted descriptive analyses.

**Results:**

We identified 723,604 adult patients who met the registry inclusion criteria at any time during the study period: 631,780 with unhealthy alcohol use, 143,690 with an AUD diagnosis, and 18,985 with an alcohol-related health problem. We identified 65,064 patients who met two or more criteria. Of the 4,973,195 adult patients with at least one encounter with the health system during the study period, the prevalence of unhealthy alcohol use was 13% (631,780/4,973,195), the prevalence of AUD diagnoses was 3% (143,690/4,973,195), and the prevalence of alcohol-related health problems was 0.4% (18,985/4,973,195). The registry cohort was 60% male (n=432,847) and 41% non-White (n=295,998) and had a median age of 41 years (IQR=27). About 48% (n=346,408) had a chronic medical condition, 18% (n=130,031) had a mental health condition, and 4% (n=30,429) had a drug use disorder diagnosis.

**Conclusions:**

We demonstrated that EHR-based data collected during clinical care within an integrated health system could be leveraged to develop a registry of patients with alcohol problems that is flexible and can be easily updated. The registry’s comprehensive patient-level data over multiyear periods provides a strong foundation for robust research addressing critical public health questions related to the full course and spectrum of alcohol problems, including recovery, which would complement other methods used in alcohol research (eg, population-based surveys, clinical trials).

## Introduction

Electronic health records (EHRs) provide a platform to study many diseases and health-related issues longitudinally in diverse populations, including identification of patients with (or at-risk of developing) conditions, and tracking treatment progress and recovery. The development of EHR-based disease registries has aided health care professionals and researchers in increasing their understanding of chronic illnesses and how to manage them [1]. For example, disease registries can facilitate the coordination of care within a health system [2,3]. However, they can also enable research on treatment effectiveness and patient outcomes, complementing other methods that are costly for repeated data collection in large populations (eg, clinical trials, surveys) [4].

Despite excessive alcohol use being a significant contributor to the global burden of disease and disability [5], to our knowledge, no population-, health system–, or EHR-based registries of individuals with alcohol problems exist. In 2016, excessive alcohol use accounted for 3 million deaths worldwide (5.3% of all deaths), which was higher than that of common conditions, such as diabetes (2.8%), road injuries (2.5%), tuberculosis (2.3%), and hypertension (1.6%) [5]. Alcohol-related death rates in the United States have also increased substantially over the past decade [6], accelerating over recent years [7]. Alcohol use is a known risk factor for serious medical conditions, including pancreatitis [8], stroke [9], and breast cancer [10], and can lead to alcohol use disorder (AUD) and alcoholic liver cirrhosis [11]. Alcohol can also impact the course of disease progression, management, and treatment outcomes for a range of conditions, including diabetes [12], depression [13,14], and anxiety [15]. While the prevalence of excessive alcohol use in the general US population ranges from 6% to 28% (depending on the definition and whether individuals with an AUD diagnosis are included) [16,17], there is evidence that it is increasing [17,18]. Therefore, alcohol problems are a significant public health concern that would benefit from being the primary focus of a disease registry.

The goal of the overall project was to assemble a registry of patients with alcohol problems by leveraging comprehensive EHR-based data within Kaiser Permanente Northern California (KPNC). The registry was developed for research specifically, but with the potential for future clinical or administrative applications such as quality improvement. KPNC is an integrated health care system that provides primary and specialty care internally (including addiction medicine and psychiatry). It has a mature, fully developed Epic EHR system (Epic Systems, Verona, WI), Kaiser Permanente (KP) HealthConnect, that stores data collected throughout the full course of patient care since 2005. Additionally, KPNC has conducted over 12 million alcohol screenings among 4 million adult members since June 2013 as part of a systematic alcohol screening, brief intervention, and referral to treatment initiative in primary care [19], which adds a robust patient-reported element to clinical data recorded in the EHR. Therefore, longitudinal, multidimensional patient-level data can be obtained (including alcohol use, health service utilization, diagnoses, medications, laboratory tests, and responses to health questionnaires), providing a unique opportunity to study the onset and progression of alcohol problems, care provided during all phases (ie, follow-up, management, continuity of care), and measurable outcomes such as changes in drinking. The objective of this paper was to describe the protocol used to develop the registry and to characterize patients who met eligibility criteria, including underlying health conditions. We include our methodological approach and considerations related to the registry, which we hope will be useful to other research teams and health systems with the ability to track unhealthy alcohol use, AUDs, and alcohol-related health problems (ie, conditions that are entirely attributable to alcohol).

## Methods

### Setting

KPNC serves 4.3 million members, comprising about one-third of the population in Northern California. The membership is diverse and highly representative of the US population with access to care [20]. Membership includes enrollees from Medicaid (12%), Medicare (16%), employer-based plans, and health insurance exchanges. KPNC members have direct access to specialty care clinics, including addiction medicine and psychiatry [21].

In June 2013, KPNC implemented Alcohol as a Vital Sign, a systematic alcohol screening, brief intervention, and referral to treatment initiative, in adult primary care [19]. While the initiative is primary care-based, the EHR screening tools are available for use in outpatient medical departments. KPNC has maintained an average 87% screening rate systemwide in adult primary care. As part of the screening, patients are asked three questions about their alcohol use, including a modified version of the evidence-based National Institute on Alcohol Abuse and Alcoholism (NIAAA) single-item screening question [16] (tailored to the patient’s age and sex)—“How many times in the past three months have you had 5 or more drinks containing alcohol in a day?” (for men aged 18-65 years), or “4 or more drinks” (for all women and for men aged ≥66 years)—and two questions that are used to calculate average drinks consumed per week—“On average, how many days per week do you have an alcoholic drink?” and “On a typical drinking day, how many drinks do you have?” The EHR issues a best practice alert during a primary care visit when screening is required (ie, first visit, annually, or every six months if unhealthy alcohol use was previously reported). The medical assistant may skip these questions for a variety of reasons (eg, late appointment arrivals, forgetting), and patients may decline to respond.

### Data Sources

Registry data are leveraged from two existing data sources: Clarity (GridApp Systems, Inc) and the Virtual Data Warehouse (VDW). Clarity is the back-end database of EHR data collected in KP HealthConnect, which we used to extract alcohol screening data. The VDW is a distributed data model developed by the Health Care Systems Research Network (HCSRN) to maintain single extract, transform, and load processes that efficiently create relational tables useful for research [22]. The VDW gathers data from various EHR-based sources, including Clarity, and legacy systems that were used before the implementation of KP HealthConnect in 2005. The VDW data has been developed over many years with standardized data definitions and formats, and rigorous quality assurance.

### Objective and Aims

In collaboration with NIAAA, we defined the objective of the registry and target population and formed research aims to frame the registry’s scope. The purpose of the registry is to study the full course of alcohol problems with the flexibility to address many research questions, such as the escalation of unhealthy drinking to development of AUDs and alcohol-related health problems, and the ability to be updated with new data. The target population for the registry is adult patients diagnosed with an alcohol problem and those at risk of developing one.

### Protocol

We developed a protocol for building the registry (available upon request), following recommendations from the US Agency for Healthcare Research and Quality [4] and other disease registries [23,24], and received approval by the Institutional Review Board at KPNC. We benchmarked our approach to that of other disease registries at KPNC (eg, HIV [25], diabetes [26], cancer [27], opioid use [28]), to determine feasibility, data storage, and access. We surveyed the literature and involved KPNC physicians in psychiatry and addiction medicine to help select key data elements and clarify data definitions. We established a plan for leveraging available data by characterizing eligibility criteria for inclusion, defining the structure of the registry, and identifying core data elements and variables needed to address the research aims. We developed codebooks to define the scope of the registry (eg, diagnosis codebook of International Classification of Diseases, 9th Revision, Clinical Modification [ICD-9] and 10^th^ Revision, Clinical Modification [ICD-10] codes), which can easily be updated to extend the breadth of data that the registry captures.

#### Inclusion Criteria

We included adult patients (age ≥18 years) with unhealthy alcohol use, an active AUD diagnosis, or an alcohol-related health problem, from any department or encounter setting within the health system. The initial registry cohort includes patients who met these criteria between June 1, 2013, (when Alcohol as a Vital Sign was implemented) to May 31, 2019. The patient’s index date was the first date in which the patient met eligibility criteria during the study period.

Unhealthy alcohol use was identified using systematic alcohol screening data collected as part of Alcohol as a Vital Sign. Using NIAAA recommended drinking guidelines [16], we defined unhealthy alcohol use as exceeding either the daily (≥5 drinks/day for men aged 18-65 years, or ≥4 drinks/day for women and for men aged ≥66 years) or weekly (>14 drinks/week for men aged 18-65 years, or >7 drinks/week for women and for men aged ≥66 years) drinking limit. To determine which risk threshold to use, we used the patient’s age and EHR-assigned sex, which is directly provided by the purchaser of a health insurance plan during enrollment. For patients with unknown sex (n=270), we used their sex assigned at birth (n=45), if available, which is a patient-reported variable collected along with gender identity in the EHR. Otherwise, we imputed sex based on the patient’s age and which single-item screening question was asked (n=225). If the patient was aged 18-65 years and asked, “How many times in the past three months have you had 5 or more drinks containing alcohol in a day?” sex was imputed as male (n=106), otherwise as female (n=119).

ICD-9 and ICD-10 codes given at any encounter at KPNC or through a claim were used to identify patients with a diagnosis of an active AUD (excluding remission codes) or an alcohol-related health problem (Table 1) [29].

**Table 1:** International Classification of Diseases (ICD) codes for identification of active alcohol use disorders and alcohol-related health problems as part of inclusion criteria for the Kaiser Permanente Northern California Adult Alcohol Registry.

Disorder, ICD^a^ version, and code				Description	
**Alcohol use disorders**					
	**ICD-9**				
		291^b^		Alcohol-induced mental disorders (eg, alcohol withdrawal delirium)	
		303^b^, except 303.03 and 303.93^c^		Alcohol dependence syndrome	
		305.0^b^, except 305.03^c^		Nondependent alcohol abuse	
	**ICD-10**				
		F10.9^b^		Alcohol use, unspecified (includes alcohol-induced mental disorders)	
		F10.2^b^, except F10.21^d^		Alcohol dependence	
		F10.1^b^, except F10.11^d^		Alcohol abuse	
**Alcohol-related health problems**					
	**ICD-9**				
		357.5		Alcoholic polyneuropathy	
		425.5		Alcoholic cardiomyopathy	
		535.3^b^		Alcoholic gastritis	
		571.0-571.3		Alcoholic liver disease	
	**ICD-10**				
		G31.2		Degeneration of nervous system due to alcohol	
		G62.1		Alcoholic polyneuropathy	
		G72.1		Alcoholic myopathy	
		I42.6		Alcoholic cardiomyopathy	
		K29.2^b^		Alcoholic gastritis	
		K70^b^		Alcoholic liver disease	
		K86.0		Alcohol-induced chronic pancreatitis	

^a^ICD: International Classification of Diseases.

^b^Any (or no) additional digits.

^c^303.03, 303.93, and 305.03 are ICD-9 remission codes.

^d^F10.21 and F10.11 are ICD-10 remission codes.

#### Structure and Data Elements

**Figure 1:**
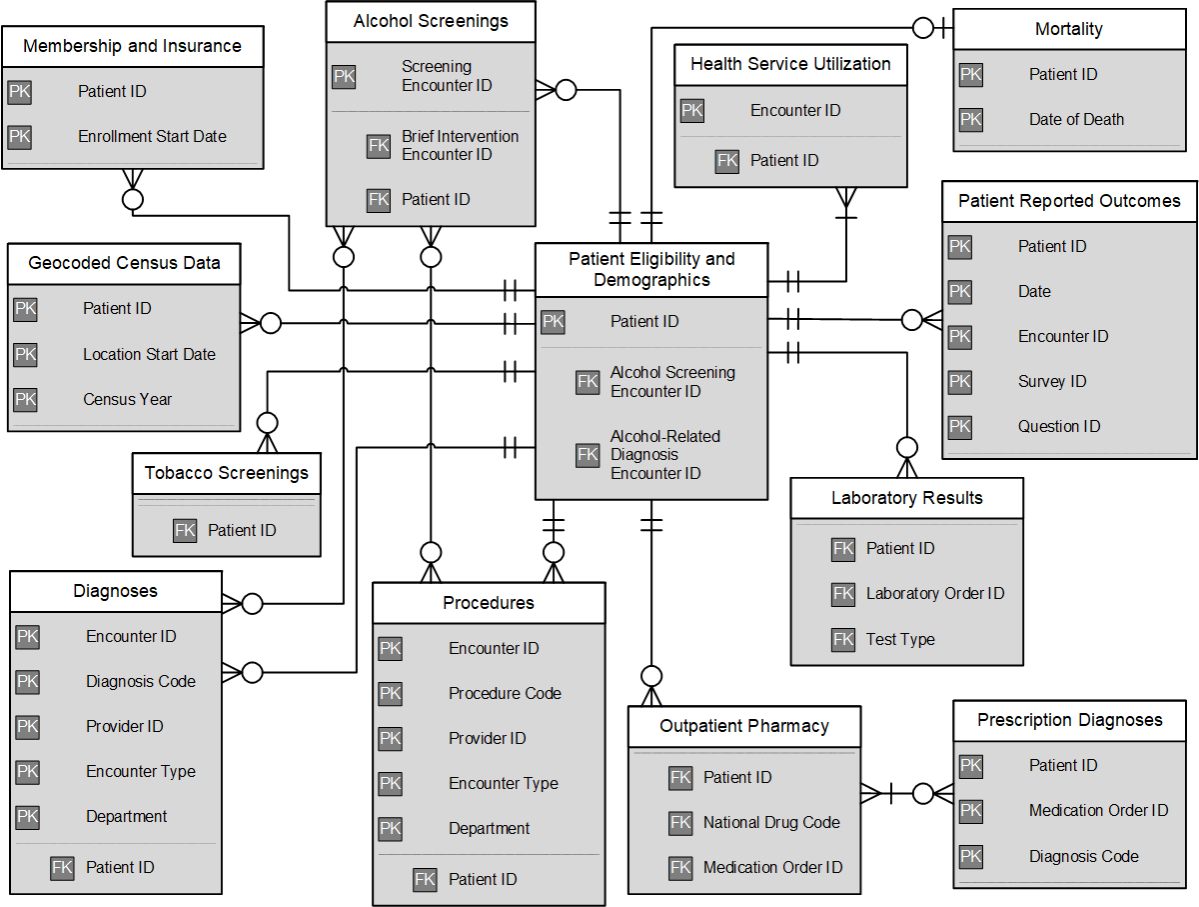
Entity-relationship diagram representing the data structure of core files in the Kaiser Permanente Northern California Adult Alcohol Registry. Primary key variables are unique identifiers that can be used with foreign key variables to link data across files. PK: primary key; FK: foreign key.

Like the VDW [22], the registry was designed as a distributed data model where each file contains one main content area, and files can be linked through key variables (eg, person ID, encounter ID; Figure 1). Main content areas include patient eligibility and demographics, alcohol screenings, membership and insurance, geocoded census data, diagnoses, procedures, outpatient pharmacy, prescription diagnoses, laboratory results, patient-reported outcomes, tobacco screenings, health service utilization, mortality, and total KPNC membership. More detailed descriptions of the data elements can be found in Multimedia Appendix 1, and specific diagnoses tracked in the registry in Multimedia Appendix 2. In each file, we retained and created variables necessary to address our research aims and used codebooks to filter the data efficiently (available upon request). We included all data from 1 year prior to the patient’s index date (serving as a time window for identifying co-occurring health conditions [30]) through the end of the study period.

### Implementation

Implementation of the protocol took about 10 months with 50% programmer time effort. We wrote programs using SAS software, version 9.4 of the SAS System for Unix (SAS Institute), to build the registry, which were designed to minimize user interaction and could be used again to refresh the registry data (eg, using macros and macro variables). We minimized data cleaning to allow future studies to make their own decisions regarding the use of the data. We created a data dictionary to describe the files and variables that comprise the registry. We also developed queries for quality control, such as identifying missing data and characterizing data storage requirements. Last, we created reporting tools to display trends of the registry data over time.

### Maintenance

Since the EHR is a constantly changing data environment, refreshing the registry with new data requires programs and documentation to be updated. For example, source variables and tables may be renamed or become deprecated during upgrades of data systems. The amount of time required to refresh the registry depends on the quantity and types of changes needed (eg, adding more ICD codes versus editing SAS programs), but may take anywhere from an hour to several days. Receiving ongoing feedback of the registry as research staff use it for their projects is also critical to ensuring the registry’s validity and usefulness.

### Analysis of the Registry Cohort

We calculated the prevalence of alcohol problems among all adult KPNC patients who had at least one encounter with the health system between June 1, 2013, and May 31, 2019. We conducted descriptive analyses to describe demographic, clinical (eg, medical and mental health conditions), and insurance characteristics of the registry cohort. We included only key variables in the current analysis to compare the registry cohort to those in other published studies. All characteristics, such as age, were based on the patient’s index date. We estimated patients’ household income and education using US Census data that has been geocoded to patients’ closest residential addresses in the year prior to and including the month of their index date. If the index date was before January 1, 2017, we used the 2010 US Census data; otherwise, we used 2017 data, since census block boundaries can change over time [31]. We used the median household income of the census block to estimate patients’ household income and categorized patients into groups used in prior epidemiologic studies of the general US population [18,32]. The education level with the highest proportion of households in the census block was used to estimate patients’ education. To identify smoking status, we used the closest screening in the year prior to and including the month of the index date. We calculated the Charlson comorbidity score, which estimates the 1-year mortality risk based on a weighted score of 17 medical conditions [33], and identified chronic medical and mental health conditions and substance use disorder diagnoses in the year prior to the index date. All analyses were conducted using SAS software version 9.4.

## Results

We identified 723,604 adult patients eligible for inclusion in the registry between June 1, 2013, to May 31, 2019: 631,780 with unhealthy alcohol use, 143,690 with an AUD diagnosis, and 18,985 with an alcohol-related health problem, anytime during the study period. Counts are not independent, as 65,064 patients met two or more eligibility criteria. Of 4,973,195 adult KPNC patients with at least one encounter with the health system during the study period, the prevalence of unhealthy alcohol use was 13% (631,780/4,973,195), the prevalence of AUD diagnoses was 3% (143,690/4,973,195), and the prevalence of alcohol-related health problems was 0.4% (18,985/4,973,195).

The registry cohort was about 60% (n=432,847) male and 40% (n=290,755) female, and there were 2 patients with other/unknown sex. In regard to gender, 0.1% (n=688) of the cohort were gender minorities (transgender, nonbinary, or other gender). The median age was 41 years (IQR=27; Table 2). The cohort was 19% (n=138,925) Latino/Hispanic, 11% (n=76,197) Asian, Native Hawaiian or Pacific Islander, and 7% (n=50,601) Black. Based on geocoded US Census data, 57% (n=409,004) of the cohort had higher household incomes (≥$70,000) and 72% (n=517,624) had some college or higher education. Most of the cohort had commercial insurance (87%, n=561,620), although 3% (n=19,834) had Medicaid. Patients had a median of 21 months (IQR=39) of follow-up data and up to 15 alcohol screenings after entering the registry (Table 2). About 48% (n=346,408) of the cohort had a chronic medical condition, 18% (n=130,031) had a mental health condition, and 4% (n=30,429) had a drug use disorder diagnosis (Table 3). The most common conditions were hypertension (21%, n=152,928), hyperlipidemia (19%, n=134,705), nicotine use disorder (12%, n=86,540), mood disorder (11%, n=82,059), anxiety disorder (11%, n=76,444), and gastroesophageal reflux (10%, n=71,159).

**Table 2:** Characteristics of patients meeting eligibility criteria for the Kaiser Permanente Northern California Adult Alcohol Registry between 6/1/2013 and 5/31/2019 (N=723,604).

Characteristic			Value
**Sex, n (%)^a^**			
	Male		432,847 (59.8)
	Female		290,755 (40.2)
	Other/Unknown		2 (<0.1)
**Gender, n (%)^a^**			
	Male		432,614 (59.8)
	Female		290,302 (40.1)
	Transgender male		217 (<0.1)
	Transgender female		241 (<0.1)
	Non-binary		229 (<0.1)
	Other/Unknown		1 (<0.1)
Age in years, median (IQR)			41.0 (27.0)
**Age group (years), n (%)^a^**			
	18-34		279,276 (38.6)
	35-49		187,072 (25.9)
	50-64		156,250 (21.6)
	≥65		101,006 (14.0)
**Race/ethnicity, n (%)^a^**			
	White		427,606 (59.1)
	Asian/Native Hawaiian/Pacific Islander		76,197 (10.5)
	Black		50,601 (7.0)
	Latino/Hispanic		138,925 (19.2)
	Native American		7,015 (1.0)
	Other/Unknown		23,260 (3.2)
**Household income (US$)^b^, n (%)^a^**			
	0-19,999		5,694 (0.8)
	20,000-34,999		38,534 (5.3)
	35,000-69,999		264,638 (36.6)
	≥70,000		409,004 (56.5)
	Unknown		5,734 (0.8)
**Education^c^, n (%)^a^**			
	Less than high school		32,446 (4.5)
	High school graduate		171,132 (23.6)
	Some college or higher		517,624 (71.5)
	Unknown		2,402 (0.3)
**Smoking status, n (%)^a^**			
	Never or former		552,618 (76.4)
	Current		115,557 (16.0)
	Unknown		55,429 (7.7)
**Charlson comorbidity score, n (%)^a^**			
		0	614,422 (84.9)
		1	64,420 (8.9)
		≥2	44,762 (6.2)
**Type of insurance, n (%)^a^**			
		None	30,033 (4.2)
		Medicaid	19,834 (2.7)
		Medicare	105,393 (14.6)
		Commercial	561,620 (77.6)
		Other	6,724 (0.9)
Enrolled via California Affordable Care Act exchange, n (%)^a^			44,110 (6.1)
Months of follow-up data in the registry, median (IQR)			21.0 (39.0)
Number of alcohol screenings, minimum-maximum			0-15

^a^Percentages may not add up to 100% due to rounding error.

^b^Median household income from geocoded census blocks to patients’ residential addresses was used as a proxy of individual-level data.

^c^The proportion of individuals within a census block with a level of education was used to estimate each patient’s education level.

**Table 3:** Diagnoses^a^ of patients in the Kaiser Permanente Northern California Adult Alcohol Registry (N=723,604).

Condition				Value, n (%)
**Chronic medical conditions**				
	Any chronic medical condition			346,408 (47.9)
	Arthritis and other rheumatic conditions			70,371 (9.7)
	Asthma			65,073 (9.0)
	Atherosclerosis			12,751 (1.8)
	Atrial fibrillation			49,141 (6.8)
	Cerebrovascular disease			14,920 (2.1)
	Chronic kidney disease			23,253 (3.2)
	Chronic liver disease			21,363 (3.0)
	Chronic obstructive pulmonary disease			21,953 (3.0)
	Chronic pain			41,089 (5.7)
	Coronary disease			20,644 (2.9)
	Dementia			2,143 (0.3)
	Diabetes			45,988 (6.4)
	Epilepsy			5,050 (0.7)
	Gastroesophageal reflux			71,159 (9.8)
	Heart failure			8,342 (1.2)
	HIV			2,424 (0.3)
	Hyperlipidemia			134,705 (18.6)
	Hypertension			152,928 (21.1)
	Migraine			23,600 (3.3)
	Osteoarthritis			66,800 (9.2)
	Osteoporosis and osteopenia			18,626 (2.6)
	Parkinson’s disease			713 (0.1)
	Peptic ulcer			3,074 (0.4)
	Rheumatoid arthritis			3,179 (0.4)
**Mental health conditions**				
	Any mental health condition			130,031 (18.0)
	**Anxiety disorder**			76,444 (10.6)
		Obsessive-compulsive disorder		1,700 (0.2)
		Panic disorder		7,823 (1.1)
		Posttraumatic stress disorder		5,312 (0.7)
	**Eating disorder**			924 (0.1)
		Anorexia nervosa		276 (<0.1)
		Bulimia nervosa		699 (0.1)
	**Mood disorder**			82,059 (11.3)
		Bipolar disorder		9,162 (1.3)
		Depression		75,445 (10.4)
		Other mood disorder		842 (0.1)
	Pervasive developmental disorder			221 (<0.1)
	**Psychoses**			6,016 (0.8)
		Schizoaffective disorder		1,427 (0.2)
		Schizophrenia		1,534 (0.2)
		Other psychoses		4,555 (0.6)
	Trauma- and stressor-related disorders			12,158 (1.7)
**Substance use disorder**				
	Nicotine use disorder			86,540 (12.0)
	**Any drug use disorder**			30,429 (4.2)
			Cannabis	15,175 (2.1)
			Cocaine	4,980 (0.7)
			Opioid	5,934 (0.8)
			Other drugs	10,418 (1.4)
			Stimulants	7,293 (1.0)

^a^Diagnoses were identified using ICD codes given at encounters in the year before the patient’s eligibility date for the registry (ie, index date).

## Discussion

In an integrated health system, we identified a large, population-based cohort of adult patients with unhealthy alcohol use, an AUD, or an alcohol-related health problem that had about 2 years of follow-up time. The KPNC Adult Alcohol Registry can evaluate the full course of alcohol problems, longitudinally and comprehensively, including early identification, initiation and engagement in treatment (including psychiatry, addiction medicine, and pharmacotherapy), and long-term outcomes (eg, drinking, physical and mental well-being), which are critical to understanding recovery. The prevalence of unhealthy alcohol use was 13%, which falls within the range reported by prior studies of the general US population (6%-28%) [16,17]. However, the prevalence of AUD diagnoses in our population (3%) was lower than the 2012-2013 prevalence of Diagnostic and Statistical Manual of Mental Disorders-5 (DSM-5) AUD (13.9% [32]) and DSM-IV AUD (12.6%, [18]) estimated from surveys of the general US population, which might be because diagnoses in health systems depend on clinician assessment and diagnosis during utilization of health care services. Only about 7.6% of individuals with AUD in the general US population seek treatment [34] Additionally, these were crude estimates of prevalence over 6 years and not standardized rates, which a future study could evaluate.

Similar to other studies using population-based survey data that indicated a higher prevalence of unhealthy drinking and AUDs in younger males [18,32], our cohort included more males than females, and younger patients (18-34 years) compared to other age groups. The registry cohort was ethnically diverse, but less representative of lower socioeconomic statuses than samples based on the general US population [18,35]. The cohort included patients with a range of mental health conditions and other substance use disorders, enabling future studies to evaluate the treatment and long-term measurable outcomes in these clinically relevant subgroups.

This EHR-based registry provides a strong foundation for robust research examining the development of alcohol problems and recovery from them. In contrast to national population-based surveys such as the National Epidemiologic Survey on Alcohol and Related Conditions (NESARC) [36] and clinical trials such as Project MATCH [37] and COMBINE [38] that collect data from participants at study visits (ie, primary data), the registry takes advantage of data that is collected during health care delivery (ie, secondary data). Primary data collection can be costly for both researchers and participants, especially in large populations, while the use of secondary data can be a cost-effective way to achieve similar research goals. Costs of an EHR-based registry include the initial investment to build it and those related to maintaining it over time, which are less than what a primary research study with equivalent sample size and time points would cost. In many ways, EHR data in KPNC are similar to that in the Veterans Health Administration (VA), the nation’s most extensive integrated health care system, which implemented alcohol screening in 2004 [39]; however, our registry cohort of KPNC members is more generalizable to the insured US population since the VA samples are predominantly male, white, and older [40,41].

Additionally, our registry data are longitudinal, spanning over 6 years as of May 31, 2019, and the registry can be continually refreshed with new data extracted from the EHR, including adding new cases and more time points for existing cases. Some current alcohol research studies utilize longitudinal data (eg, NESARC, Project MATCH), but many are repeated cross-sectional studies with different samples (eg, National Health and Nutrition Examination Survey [42], National Health Interview Survey). The registry data are also comprehensive, capturing not only a variety of diagnoses and lab tests that can be used to measure physical functioning, but also health service utilization, insurance factors, and patient-reported outcomes, including alcohol use levels.

We included gender minorities in our registry, given recent research demonstrating a high prevalence of unhealthy drinking in this population [43]. Additionally, the NIAAA has recognized that transgender communities are relevant subpopulations to consider for addressing health disparities [44]. However, there are no general guidelines for how gender minorities should be screened and which risk thresholds to use. For purposes of this registry, we used the patient’s EHR-assigned sex, and when applicable, their response to sex assigned at birth to determine unhealthy alcohol use. We present this approach to be transparent in how sex and gender were operationalized in our registry with the hope of strengthening future research in this area [43].

### Limitations

EHR-based registries enable observational studies of “real-world” settings (eg, comparative effectiveness research), an alternative to randomized controlled trials, which may not be feasible; however, using secondary data for research has limitations, including the omission of essential variables and potential for bias (eg, selection bias, information bias, confounding). For example, clinicians in addiction medicine and psychiatry assess AUD symptoms based on the DSM-5, but detailed data are not entered in the EHR. Instead, clinicians record ICD codes to indicate AUD diagnoses, which we use in the registry. While ICD codes for AUDs are not given lightly in other departments, they do occur, and it is not clear what guidelines are used. Therefore, a future validation study of alcohol screening results and the use of these ICD codes is warranted. We also do not have direct measures of individual socioeconomic status (eg, income, education), which are important factors associated with unhealthy alcohol use [18], or social functioning (eg, the Psychosocial Functioning Inventory [45]), an important recovery outcome [46]. Though not only an issue with secondary data analysis, missing data can create bias in a study if it is not missing completely at random; therefore, future studies utilizing the registry data should check for missingness and apply proper statistical methods to address issues as needed [47]. Reliance on accurate reporting of alcohol use and other measures is also a concern; however, it is not a unique problem of EHR data and shared by other studies that collect self-report data. While novel statistical methodologies can be applied to deal with issues of confounding [48] (eg, the counterfactual framework [49]), temporality may remain an issue. Measures of alcohol use and diagnoses are recorded in the EHR when patients seek care rather than when alcohol-related issues emerge, similar to other disease-based registries that rely on data collected during care (eg, diagnostic tests for cancer) and survey-based studies that gather data on past-year or lifetime alcohol problems without specific dates.

Sex and gender variables in the EHR can change and are not collected longitudinally, so their values in the registry reflect what was present at the time of the data extraction rather than historical values, for example, at the time of alcohol screening. We are also not certain which variables are used to determine the appropriate screening questions and risk thresholds (especially for gender minorities), which a future study could evaluate. Therefore, some alcohol screening results may have been misclassified in the registry, affecting eligibility; however, we expect this to have a minimal impact on future studies.

### Future Directions

While we included only core data elements that were necessary to address our research aims, the registry could be extended to include other types of data, including provider information, family members of patients with alcohol problems, and medications prescribed off-label to treat AUD (eg, gabapentin [50]). Other health systems in the HCSRN with harmonized VDW data [22] may also want to create their own registry of alcohol problems, enabling the potential for multi-site studies [51-53]. The registry’s utility may also extend beyond research to clinical or administrative purposes, for example, to manage care or evaluate performance, which would require additional support from KPNC organizational stakeholders and institutional review boards to protect patient privacy and confidentiality.

### Conclusions

We demonstrate that EHR-based data collected during routine clinical care within an integrated health care system can be leveraged to develop a registry of patients with alcohol problems that is flexible and can be easily refreshed and extended. The registry can be used to address critical public health questions related to the full spectrum and course of alcohol problems, which will complement other methods used in alcohol research. Future analyses will aim to provide insight on how to strengthen efforts in the prevention of alcohol-related disability and mortality and improve patient-centered health care delivery. We hope that other researchers and health systems interested in assembling a similar registry can take advantage of the time we invested in developing this protocol.

## Appendix

Multimedia Appendix 1 Detailed descriptions of data elements in the Kaiser Permanente Northern California Adult Alcohol Registry.

Multimedia Appendix 2 Additional diagnoses tracked among patients in the Kaiser Permanente Northern California Adult Alcohol Registry.

## Abbreviations

AUD: alcohol use disorder
EHR: electronic health record
HCSRN: Health Care Systems Research Network
ICD: International Classification of Diseases
ICD-9: International Classification of Diseases, 9th Revision, Clinical Modification
ICD-10: International Classification of Diseases, 10th Revision, Clinical Modification
KP: Kaiser Permanente
KPNC: Kaiser Permanente Northern California
NESARC: National Epidemiologic Survey on Alcohol and Related Conditions
NIAAA: National Institute on Alcohol Abuse and Alcoholism
VDW: Virtual Data Warehouse
