# Supplementary material for: The Kaiser Permanente Northern California Adult Alcohol Registry, an Electronic Health Records-Based Registry of Patients With Alcohol Problems: Development and Implementation
Source: JMIR Med Inform. 2020 Jul 22;8(7):e19081. doi: 10.2196/19081 (PMC7407243; doi:10.2196/19081)
Supplement: Multimedia Appendix 1 [file medinform_v8i7e19081_app1.docx]

## Multimedia Appendix 1

Detailed descriptions of data elements in the Kaiser Permanente Northern California Adult Alcohol Registry.

Patient Eligibility and Demographics

This is the main file that links to all other files, containing patient demographic information (e.g., EHR-assigned sex, gender, race/ethnicity, age at index date) and data related to eligibility (i.e., an alcohol screening indicating unhealthy alcohol use and/or an AUD or alcohol-related health problem diagnosis). We compared patient responses to gender identity and sex assigned at birth documented in the EHR to create a gender variable. If responses differed, then patients were coded as transgender female, transgender male, non-binary, or other, otherwise as female or male. If both variables were unknown, then gender was assumed to be the EHR-assigned sex.

Alcohol Screenings

All alcohol screenings conducted during the study period are contained in this file. We determined whether each screening was positive for unhealthy alcohol use based on the NIAAA guidelines for exceeding daily and/or weekly drinking limits [1]. We also determined whether a brief intervention was conducted within 60 days of each screening (based on codes in the diagnosis and procedure files, see below). We chose 60 days to be as flexible as possible for identifying brief interventions; however, future studies using the data may choose to place further restrictions.

Membership and Insurance

All membership data is stored in this file, such as enrollment dates, types of insurance (e.g., Medicare, Medicaid, commercial), and enrollment mechanisms (e.g., California Affordable Care Act exchange).

Geocoded Census Data

This file contains U.S. Census 2010 and 2017 data that have been geocoded to patients’ residential addresses [2,3]. Since socioeconomic data are limited in EHRs, census block-level data (e.g., median household income, proportion of adults with different levels of education) can be used as a proxy for individual-level data.

Diagnoses

All diagnoses made at an encounter with the health system that are in the *ICD* codebook (available upon request) are contained in this file. In addition to tracking diagnosis codes for AUDs and alcohol-related health problems [4] (Table 1), we also included codes to track other substance use disorders [5], common chronic medical and mental health conditions [6,7], and substance-abuse related medical conditions [8] (Multimedia Appendix 2). We also included *ICD* codes used for brief alcohol interventions (*ICD-9*: V65.42, V65.49; *ICD-10*: Z71.41, Z71.89).

Procedures

This file contains brief alcohol interventions based on Current Procedural Terminology (96160, 99420, 99408, 99409) and Healthcare Common Procedure Coding System codes (G0396, G0397, G0443, H0050), which are in a procedure codebook.

Outpatient Pharmacy

Information on outpatient prescriptions dispensed from KPNC pharmacies, such as generic and brand names, number of units dispensed, and days of supply, are included in this file. For the registry, only medications listed in the pharmacy codebook are included (available upon request), which include those used to treat AUDs (i.e., acamprosate, disulfiram, naltrexone, and topiramate) [9–11], nicotine use disorders [12], opioid use disorders [13], psychiatric disorders (i.e., antidepressants, mood stabilizers, sedative-hypnotics, anxiolytics, and antipsychotics) [14–16], and common medical conditions (i.e., diabetes, hypertension, hypoglycemia) [17–19]. Psychiatric and addiction medicine physicians (M.E.H. and M.Z.G., respectively) reviewed the medications, along with their classifications, to ensure validity.

Prescription Diagnoses

Diagnoses can be associated with each prescription order using *ICD* codes, which are contained in this file.

Laboratory Results

Results from common laboratory tests to measure functioning of the liver [20,21], glycemic control [22], and cholesterol levels [23] are included.

Patient-Reported Outcomes

Patients in KPNC primary care and psychiatric outpatient clinics are prompted to complete the 9-item Patient Health Questionnaire and 2-item Generalized Anxiety Disorder questionnaire in a combined survey to screen for depression and anxiety and to track treatment progress.

Tobacco Screenings

Current and former tobacco use are routinely asked by medical assistants at each primary care encounter and entered in the EHR.

Health Service Utilization

All outpatient, inpatient, and emergency department encounters are captured in this file. Outpatient encounters are further categorized by department, including addiction medicine, psychiatry, and primary care.

Mortality

Date of death from the EHR and National Death Index [24] are stored in this file.

Total KPNC Membership (Person-Time)

Among the entire KPNC population, we totaled the number of patient members by service area, gender, age group, and race/ethnicity for each month, quarter, and year of the registry period. These counts are estimates of the total person-time at risk of developing an alcohol problem, which can be used as denominators in calculating rates (e.g., rate of identifying new eligible cases for inclusion in the registry).

## References

1. National Institute on Alcohol Abuse and Alcoholism. Helping patients who drink too much: a clinician’s guide. 2005. Available at: https://pubs.niaaa.nih.gov/publications/Practitioner/CliniciansGuide2005/guide.pdf. Accessed March 13, 2018.

2. U.S. Census Bureau. American FactFinder. 2018. Available at: https://factfinder.census.gov/faces/nav/jsf/pages/index.xhtml?. Accessed March 13, 2019.

3. Young-Wolff KC, Klebaner D, Campbell CI, et al. Association of the Affordable Care Act with smoking and tobacco treatment utilization among adults newly enrolled in health care. Med Care 2017;55(5):535–541. PMID:28288073

4. Centers for Disease Control and Prevention. Alcohol-Related ICD Codes. Alcohol and Public Health: Alcohol-Related Disease Impact (ARDI). Available at: https://nccd.cdc.gov/DPH_ARDI/Info/ICDCodes.aspx. Accessed January 13, 2019.

5. Campbell CI, Parthasarathy S, Altschuler A, Young-Wolff KC, Satre DD. Characteristics of patients with substance use disorder before and after the Affordable Care Act. Drug Alcohol Depend 2018 01;193:124–130. PMID:30366189

6. Ornstein SM, Nietert PJ, Jenkins RG, Litvin CB. The prevalence of chronic diseases and multimorbidity in primary care practice: a PPRNet report. J Am Board Fam Med 2013 Oct;26(5):518–524. PMID:24004703

7. Bower P, Gilbody S. Managing common mental health disorders in primary care: conceptual models and evidence base. BMJ 2005 Apr 9;330(7495):839–842. PMID:15817554

8. Weisner C, Mertens J, Parthasarathy S, Moore C, Lu Y. Integrating primary medical care with addiction treatment: a randomized controlled trial. JAMA 2001 Oct 10;286(14):1715–1723. PMID:11594896

9. U.S. Department of Health and Human Services. Medication for the Treatment of Alcohol Use Disorder: A Brief Guide. HHS Publication No. (SMA) 15-4907. Rockville, MD; 2015 Available at: https://store.samhsa.gov/sites/default/files/d7/priv/sma15-4907.pdf. Accessed May 2, 2019.

10. Center for Substance Abuse Treatment. Chapter 4—Oral Naltrexone. Rockville, MD: Substance Abuse and Mental Health Services Administration (US); 2009. Available at: https://www.ncbi.nlm.nih.gov/books/NBK64042. Accessed May 2, 2019.

11. Paparrigopoulos T, Tzavellas E, Karaiskos D, Kourlaba G, Liappas I. Treatment of alcohol dependence with low-dose topiramate: an open-label controlled study. BMC Psychiatry 2011 Mar 14;11:41. PMID:21401921

12. Ebbert JO, Wyatt KD, Hays JT, Klee EW, Hurt RD. Varenicline for smoking cessation: efficacy, safety, and treatment recommendations. Patient Prefer Adherence 2010 Oct 5;4:355–362. PMID:21049087

13. U.S. Department of Health and Human Services. Medications for Opioid Use Disorder. HHS Publication No. (SMA) 18-5063FULLDOC. Rockville, MD: Substance Abuse and mental Health Services Administration; 2018. Available at: https://store.samhsa.gov/system/files/sma18-5063fulldoc.pdf. Accessed May 10, 2019.

14. Public Resources from the Mental Health Research Network: MHResearchNetwork/MHRN-Central. MHResearchNetwork; 2019. Available at: https://github.com/MHResearchNetwork/MHRN-Central. Accessed July 24, 2019.

15. John M. Eisenberg Center for Clinical Decisions and Communications Science. Antipsychotic Medicines for Treating Schizophrenia and Bipolar Disorder: A Review of the Research for Adults and Caregivers. Comparative Effectiveness Review Summary Guides for Consumers Rockville (MD): Agency for Healthcare Research and Quality (US); 2005 Accessed May 29, 2019. PMID:23741769

16. Center for Medicare & Medicaid Services. Atypical Antipsychotics: U.S. Food and Drug Administration-Approved Indications and Dosages for Use in Adults. Available at: https://www.cms.gov/Medicare-Medicaid-Coordination/Fraud-Prevention/Medicaid-Integrity-Education/Pharmacy-Education-Materials/Downloads/atyp-antipsych-adult-dosingchart.pdf. Accessed May 29, 2019.

17. Ganesan K, Sultan S. Oral Hypoglycemic Medications. StatPearls Treasure Island (FL): StatPearls Publishing; 2019. PMID:29494008

18. Luna B, Feinglos MN. Oral Agents in the Management of type 2 diabetes mellitus. AFP 2001 May 1;63(9):1747.

19. Hu J, Deng A, Zhao Y. Ertugliflozin as a monotherapy for the treatment of type 2 diabetes. Expert Opin Pharmacother 2018 Nov;19(16):1841–1847. PMID:30223693

20. Gowda S, Desai PB, Hull VV, et al. A review on laboratory liver function tests. Pan Afr Med J 2009 Nov 22;3. PMID:21532726

21. Niemelä O, Nivukoski U, Bloigu A, et al. Laboratory test based assessment of WHO alcohol risk drinking levels. Scand J Clin Lab Invest 2019 Apr;79(1–2):58–64. PMID:30721633

22. National Committee for Quality Assurance. Comprehensive Diabetes Care. HEDIS Measures and Technical Resources. Available at: https://www.ncqa.org/hedis/measures/comprehensive-diabetes-care. Accessed September 24, 2019.

23. Centers for Disease Control and Prevention. Getting Your Cholesterol Checked. 2019. Available at: https://www.cdc.gov/cholesterol/cholesterol_screening.htm. Accessed August 7, 2019.

24. Centers for Disease Control and Prevention. National Death Index. National Center for Health Statistics. 2020. Available at: https://www.cdc.gov/nchs/ndi/index.htm. Accessed February 27, 2020.
