# Supplementary material for: The Kaiser Permanente Northern California Adult Alcohol Registry, an Electronic Health Records-Based Registry of Patients With Alcohol Problems: Development and Implementation
Source: JMIR Med Inform. 2020 Jul 22;8(7):e19081. doi: 10.2196/19081 (PMC7407243; doi:10.2196/19081)
Supplement: Multimedia Appendix 2 [file medinform_v8i7e19081_app2.docx]

| **Multimedia Appendix 2**. Additional diagnoses tracked among patients in the Kaiser Permanente Northern California Adult Alcohol Registry. ^a^ | |
| --- | --- |
| **Substance use disorders ^b^** | cannabis, cocaine, hallucinogen, inhalant, multi-drug, nicotine, opioid, other, sedative/hypnotic/anxiolytic, stimulant |
| **Chronic medical conditions** | arthritis and other rheumatic conditions, asthma, atrial fibrillation, atherosclerosis, cerebrovascular disease, chronic kidney disease, chronic liver disease, chronic obstructive pulmonary disease, chronic pain, coronary disease, dementia, diabetes, epilepsy, gastroesophageal reflux, heart failure, HIV, hyperlipidemia, hypertension, migraine, osteoarthritis, osteoporosis, Parkinson’s disease, peptic ulcer, rheumatoid arthritis |
| **Mental health conditions** | anxiety disorders (obsessive compulsive disorder, panic disorder, post-traumatic stress disorder), eating disorders (anorexia nervosa, bulimia nervosa), mood disorders (bipolar disorder, depression, other), pervasive developmental disorders, psychoses (schizoaffective disorder, schizophrenia, other), trauma- and stressor-related disorders |
| **Substance abuse-related medical conditions** | acid-related disorders, anxiety and nervous disorders, asthma, chronic obstructive pulmonary disease, depression, diseases of the pancreas, alcohol and drug use complicating mother-childbirth, drug neuropathy, excess blood alcohol level, hepatitis C, hypertension, injury and poisonings, ischemic heart disease, liver cirrhosis, pneumonia, poisoning by alcohol, psychoses, toxic effects of alcohol |
| ^a^ Additional to diagnoses in Table 1.  ^b^ Substance use disorders in remission are also tracked. | |
